# Supplementary material for: Multifaceted Elevation of ROS Generation for Effective Cancer Suppression
Source: Nanomaterials (Basel). 2022 Sep 11;12(18):3150. doi: 10.3390/nano12183150 (PMC9502709; doi:10.3390/nano12183150)
Supplement: Supplementary file 1 [file nanomaterials-12-03150-s001.zip › nanomaterials-1843165-supplementary.pdf]

supplementary materials

Multifaceted Elevation of ROS Generation for Effective Cancer Suppression

Huizhe Wang [1,2,†], Mengyuan Cui [2,†], Yanqi Xu [2], Tianguang Liu [2], Yueqing Gu [2], Peng Wang [2] and Hui Tang [1,*]

[1] Stem Cell Clinical Research Center, Shandong Provincial Hospital Affiliated to Shandong First Medical University, Jinan 250021, China
[2] Department of Biomedical Engineering, School of Engineering, China Pharmaceutical University, Nanjing 210009, China

* Correspondence: tanghui1110@163.com
† The authors contributed equally to this paper

Experimental Section

Materials

All reagents and chemicals were obtained from commercial corporations. Anhydrous dichloromethane and dimethyl sulfoxide (DMSO) were supplied by Hiens Biochemical Company (Tianjin, China). 1-Ethyl-(3-dimethylamino propyl) carbodiimide hydrochloride (EDCI), 4-dimethylamino pyridine (DMAP), and dimethyl sulfoxide were purchased from Anaiji. Methanol, urea, acetic acid, ethyl acetate, and petroleum ether were purchased from Nanjing Chemical Reagent Co., Ltd. *N*,*N*-dimethyl formamide (DMF), *N*-hydroxysuccinimide (NHS), bovine serum albumin (BSA), and lonidamine (LND) was obtained from Aladdin Biotechnology Co., Ltd (Shanghai, China). 4% paraformaldehyde fixative, penicillin, streptomycin, DMEM-H medium, NMEM medium, 1640 medium, fetal bovine serum, Annexin V-FITC apoptosis detection kit, 3-(4,5-two Methylthiazol-2-yl)-2,5-diphenyltetrazolium ammonium bromide (MTT), SDS-PAGE gel kit, and Hoechst 33342 nuclear staining kit were purchased from Jiangsu Kaiji Biotechnology Co., Ltd. The JC-1 mitochondrial membrane potential probe was purchased from Xi'an Ruixi Biotechnology Co., Ltd. The H_2O_2 detection kit and ROS detection kit were purchased from Nanjing Jiancheng Biotechnology Co., Ltd. The human breast cancer cell line MCF-7 and the human liver cancer cell HepG2 were provided by Nanjing KGI Biotechnology Co., Ltd. Crystal Violet was supplied by Wokai Biotechnology (Beijing) Co., Ltd. A transwell cell with the 8 μm pore size was obtained from Corning. Various rabbit-derived antibodies, including PINK1 antibody and Prx3 antibody, were purchased from Shenyang Wanlai Biotechnology Co., Ltd. The GAPDH rabbit monoclonal antibody was provided by Biyuntian Biotechnology Company (Nanjing). All other reagents were purchased from commercial sources and used and stored by the instructions.

Human breast cancer cell line MCF-7 was obtained from Nanjing KGI Biotechnology Co., Ltd. (Nanjing, China). Female immunodeficiency mice (17-21 g) were purchased from Nanjing Qinglongshan Animal Farm.

All the animal procedures were performed by the Guidelines for Care and Use of Laboratory Animals of China Pharmaceutical University and experiments were approved by China Pharmaceutical University Animal Care and Use Committee.

Instruments

Analytical balance (BT25S, Beijing Sartorius Instrument System Co., Ltd., China) was used for weighing solid materials. The constant temperature water bath (Changzhou Guohua Electric Co., Ltd., China) and temperature-controlled magnetic stirrer (Vortex Mixer V6, Essenscien, USA) were employed for compound synthesis. The absorbance was measured by an ultraviolet-visible spectrophotometer (UV-2550, Shimadzu, Japan). Fluorescence Spectroscopy was performed by a Fluorescence spectrophotometer (F97 Pro, Shanghai Lingguang, China). The average diameter of the prepared nanoparticles was detected by dynamic laser light scattering (DLS, Zetasizer Nano ZS90, Malvern). The

Bruker Advance-300 spectrometer (Bruker, Germany) was employed to obtain ^1H NMR spectra. Confocal laser scanning fluorescence microscope (FluoView TM, FV1000, Olympus, Japan) was carried out for cell and tissue imaging. Cell and Ca2+ fluorescence images were tested by an inverted fluorescence microscope (DMi8, Leica, Germany). Cell apoptosis was detected by flow cytometry (Becton, Dickinson and Company, USA). Small animal imaging was performed by a near-infrared small animal imaging system (SI Imaging AmiX, Spectral Instruments Imaging, USA).

Synthesis of g-C_3N_4

g-C_3N_4 was synthesized according to the literature method [1]. Typically, a mixture of urea (10 g), NH_4Cl (15.5 g), and 30% H_2O_2 (20 mL) were added into a 100 mL beaker and stirred for 10 min, then the white solid was obtained and transferred to oxidation in an aluminum crucible. After being calcined in a muffle furnace at 550 °C for 4 hours, the product was reduced to room temperature, then the crude was washed with H_2O/ethanol three times and placed in an oven at 70 °C for drying 12 hours to generate g-C_3N_4 as yellow solid.

Synthesis of MLND and characterization

The corresponding derivative MLND was obtained by condensing lonidamine (LND) with mitochondrial-targeted (2-aminoethyl) triphenylphosphonium Bromide. LND (321 mg, 1 mmol), HOBT (203 mg, 1.5 mmol), EDCI (287 mg, 1.5 mmol), DMAP (98 mg, 0.8 mmol) were dissolved in 100 mL a bottom flask containing 30 mL anhydrous dichloride and stirred at room temperature for 20 min, then (2-aminoethyl) triphenylphosphonium bromide (386 mg, 1 mmol) was added to the above mixture and continue stirring for 4 h at the same temperature. At the same time, the process was monitored by thin-layer chromatography under fluorescent lamps at 254 and 365 nm until it was completed, then the solvent was evaporated and the crude was purified by silica gel column chromatography with dichloromethane and methanol (v:v = 10:1) to harvest product MLND as white power (yield: 82%). ^1H NMR (500 MHz, $CDCl_3$) δ 8.93 (s, 1H), 8.22 (d, J = 8.1 Hz, 1H), 7.89 (dd, J = 12.8, 7.8 Hz, 6H), 7.73 (t, J = 7.1 Hz, 3H), 7.66 (td, J = 7.3, 3.2 Hz, 6H), 7.42 (d, J = 8.5 Hz, 1H), 7.39-7.29 (m, 3H), 7.24 (dd, J = 14.4, 7.3 Hz, 3H), 5.69 (s, 2H), 4.15 (s, 2H), 4.03 (s, 3H). HRMS (ESI) $[M+H]^+$ Calculated for $C_{35}H_{29}Cl_2N_3OP^+$: 610.15035; Found: 610.18325.

Synthesis of Fe_3O_4-MLND@g-C_3N_4 @LOD (FGLM)

Fe_3O_4 nanoparticles (100 μL, 1 mg mL^{-1}) and distilled water (900 μL) were mixed to obtain diluent with a final concentration of 100 μg mL^{-1}, then the mixture was placed in a 10 mL EP tube. Next, g-C3N4 ethanol solution (2 mL), LOD-NH2 (1 mL, 1 mg mL^{-1}) and MLND (2 mL, 10 μM) were added. After sonicating for 15 minutes, the above mixture was stirred in the dark for 12 h, product **FGLM** was collected after being lyophilized on a freeze dryer for 24 h.

Synthesis of Fe_3O_4-MLND-LOx@g-C_3N_4@$CaCO_3$ (FGLMC)

$CaCl_2$ powder (16.6 mg) was added to a 50 mL beaker containing FGLM aqueous solution (20 mL, 200 μg mL-1), then an EP tube bearing 6 g of ammonium bicarbonate was opened and placed next to the beaker. A 500 mL beaker was buckled upside down on them to create a sealed environment. After being placed for 12 h in the dark, the mixture was centrifuged at 12000 rpm/min to obtain the final product **FGLMC**.

LOx Loading and Lactate Consumption Assay

The successful loading of LOx was verified by the sodium dodecyl sulfate-polyacrylamide gel electrophoresis (SDS-PAGE) experiment. LOx and **FGLMC** were separately mixed with 1×Loading Buffer (v:v = 1:4) and boiled for 3 min to prepare the LOx protein sample and **FGLMC** protein sample. Then the two protein samples were

loaded and electrophoresed. After staining and decolorizing, corresponding images and data were obtained.

200 μL of PBS and different concentrations (10, 20, 50, 80, 100 μg mL^{-1}) of LOx were configured, then the designated reagents were added to the 96-well plate to react according to the instructions of the H_2O_2 detection kit. The average absorbance at 240 nm (A_{240}) on the microplate reader was recorded. What's more, the concentration of H_2O_2 was calculated according to the formula below.

Formula of H_2O_2 concentration calculation: c=A/(ε×b)

c: the sample concentration (mol·L^{-1}); A: the absorbance value; ε: the wavelength-dependent molar extinction coefficient (M^{-1}·cm^{-1}), ε_{H2O2} =43.6 M^{-1} cm^{-1}; b: optical path (cm).

C_{H2O2} (M)=A_{240}/(43.6×b) or C_{H2O2} (mM)=22.94×A_{240}/b.

Cell culture

The human breast cancer cells MCF-7 were incubated on the cell culture plate in Dulbecco's Modified Eagle Medium (DMEM) containing 10% of fetal bovine serum, penicillin (100 U mL^{-1}), and streptomycin (100 U mL^{-1}) at 37 °C with 5% CO_2 in the cultivation.

Mitochondrial Targeting Study

MCF-7 cells were seeded on two 6-well plates at a density of 5×10^5 cells per well and incubated for 24 h, 100 μL of PBS, Fe_3O_4 (20 μg mL^{-1}), Fe_3O_4@g-C_3N_4 (20 μg mL^{-1}), **FGLM** (20 μg mL^{-1}), **FGLMC** (20 μg mL^{-1}) were added to the wells and cultured for 2h. After that, these cells were stained with the mitochondrial membrane potential probe JC-1 and then subjected to inverted fluorescence imaging and flow cytometry respectively.

Ca^{2+} Release of FGLMC

MCF-7 cells were incubated overnight, then PBS and FGLM (10 μg mL^{-1}), **FGLMC** (5 μg mL^{-1}), **FGLMC** (10 μg mL^{-1}), and **FGLMC** (20 μg mL^{-1}) were respectively incubated with the cells for 2 h. After which, the cell supernatant was discarded, Rhod-2 AM (5 μM) probe was added to the cells and incubated for 30 min. Then, the supernatant was removed, and the cells were washed twice with PBS (pH=7.4) and placed on an inverted fluorescence microscope for Ca^{2+} fluorescence imaging.

To determine the release of Ca^{2+} under different pH conditions, MCF-7 cells were treated according to the previous step and incubated with the medium (pH 5.0 and pH 7.4) adjusted by dilute hydrochloric acid (HCl). Then, **FGLMC** (20 μg mL^{-1}) was added to different pH media cells and incubated for 2 h. After that, the supernatant was discarded and Rhod-2 AM (5 μM) probe was added for another 30 min' incubation. The post-processing was performed in the same way as the previous step, fluorescence images of Ca^{2+} release at different pH levels were obtained.

To investigate the effect of incubation time on the release of Ca^{2+} in **FGLMC**, MCF-7 cells were also treated previously and **FGLMC** (20 μg mL-1) was added to the cells and separately incubated for 0 h, 2 h, and 3 h. The after-treatments were adopted in the same method as the above step for capturing the fluorescence images of the cells after different incubation times.

Detection of ROS

MCF-7 cells were seeded on two 6-well plates at a density of 5×10^5 cells per well and incubated overnight. One of the plates was divided into 4 groups: PBS, LND (20 μM), **FGLMC** (20 μg mL^{-1}), **FGLMC** (20 μg mL^{-1}) + 660 nm Laser (100 mW cm^{-2}, 5 min) for inverted fluorescence detection. The other plate was divided into 5 groups: PBS, LND (20 μM), MLND (20 μM), **FGLMC** (20 μg mL^{-1}), **FGLMC** (20 μg mL^{-1}) + 660 nm Laser (100 mW cm^{-2}, 5 min) for flow detection. All groups were incubated with the cells for 2 hours and then the supernatant was removed. After washing with PBS, the DCFH-DA probe was added and incubated for another 20 minutes. Finally, fluorescence imaging and flow cytometry were performed respectively after PBS washing again.

H_2O_2 Detection

MCF-7 cells were incubated for 24 h, 200 μL of PBS, and different concentrations of **FGLMC** (10, 20, 50, 80, 100 μg mL^{-1}) were separately added to the cells and cultured for another 4 h. After trypsin digestion, centrifugation was performed to collect the supernatant, and H_2O_2 lysate was further added to break the cells. Then the centrifugation was employed at a speed of 12000 rpm/min for 3-5 min at 4 °C. The H_2O_2 concentration in the cell supernatant was detected with the Hydrogen Peroxide Assay Kit.

Vitro Cytotoxicity

The cells in the logarithmic growth phase were seeded in a 96-well plate at a density of 1×10^5 cells per well, and cultured in a 37°C, 5% CO_2 incubator for 24 h. After the cells adhered to the wall, the fresh medium was joined. 200 μL of Fe_3O_4 (0, 20, 50, 80, 100, 200, 300 μg mL^{-1}), g-C_3N_4 (0, 20, 50, 80, 100, 200, 300 μg mL^{-1}) were separately added to the cells to continue the culture for another 24 h. Additionally, another group of cells was exposed to three formulations, including g-C_3N_4, FGLM, and FGLMC at different concentrations (0-20 μg mL^{-1}), respectively (n = 3). After incubation for 4 h, the cells were washed with PBS, followed by 5 min's laser treatment (660 nm, 100 mW cm^{-2}). The cells without laser treatment were used as the controls. After incubation for another 24 h, the cell viability was determined using the colorimetric MTT assay. The IC_{50} of different formulations was calculated.

Apoptosis assay

MCF-7 cells (5×10^5 cells per well) were seeded in two 6-well plates and incubated respectively with **FGLMC (**20 μg mL^{-1}), MLND (20 μM), and LND (20 μM) for 24 h. The cells were collected and stained with Annexin V-FITC and PI. One plate was directly placed under an inverted microscope for observation, and the other one was digested with trypsin without EDTA and centrifuged, then measured by flow cytometry.

MCF-7 cells (5×10^5 cells per well) were seeded in two 6-well plates. One group was treated with FGLM (20 μg mL^{-1}) and **FGLMC** (20 μg mL^{-1}). After incubation for 4 h, the medium was replaced with the fresh one and the cells were irradiated (660 nm, 100 mW cm^{-2}) for 5 min with the laser-free cells as the control. After 24 h, both Calcein AM (2 μM) and PI (2 μM) were added, followed by confocal imaging 30 min later. The excitation wavelength was 488 nm (Calcein AM) and 532 nm (PI), respectively. For the other group, the apoptosis analysis employed the Annexin V-FITC/PI probe pair. LND (20 μM), MLND (20 μM), FGLM (20 μg mL^{-1}) and **FGLMC** (20 μg mL^{-1}) were respectively treated, irradiation conditions and other details were the same as the above live/dead staining experiments. The extent of apoptosis was quantified by flow cytometry.

Migration and Invasion Assay

MCF-7 cells were Inoculated in a 6-well plate overnight according to the previous cell culture method. The cells were scratched in the middle of the well, then the medium was removed, the cells were washed twice with PBS, and a serum-free medium was added to each well. Then images of the cells were captured by utilizing the inverted fluorescence microscope according to the time point (0, 12, 24, 48 h).

MCF-7 cells were cultured in a pre-chilled serum-free medium and seeded in the upper cavity of the transwell chamber at a density of 5×10^4 cells/well. At the same time, a normal medium containing PBS, LND (20 μM), MLND (20 μM), and **FGLMC** (20 μg mL^{-1}) were separately added into the lower cavity. After culturing for 24 hours, the cells were fixed with 4% paraformaldehyde solution for 15 minutes, washed with PBS, and stained with 0.2% crystal violet for 20 minutes. Thereafter, the cells were washed again with PBS and imaged with an inverted fluorescence microscope to obtain a migration map.

Western Blotting

MCF-7 cells were incubated with PBS, LND (20 μM), MLND (20 μM) and **FGLMC** (20 μg mL^{-1}), another group of MCF-7 cells were cultured with different concentrations of

FGLMC (5, 10, 20, 50 μg mL^{-1}) under irradiation (660 nm, 100 mW cm^{-2}) for 5 min. Then the response group was lysed with RIPA lysis buffer, and the extracted protein content was semi-quantitated through the BCA Protein Assay Kit. The lysates were loaded in 8% sodium dodecyl sulfate-polyacrylamide (SDS- PAGE) gel, which was further transferred to a poly (vinylidene difluoride (PVDF) membrane. After blocking the membrane with skim milk (5% w/w) in Tris buffer saline with Tween 20 (TBST) for 2 h, the membrane was washed with TBST. Immunoblotting was employed by incubating the protein with anti-Prx3, anti-PINK1, and anti-GAPDH at 4 °C for 24 h. Afterward, the membrane was further treated by antigoat HRP secondary antibody for 2 h. Finally, Image J software was used to semi-quantitatively analyze and compare the development results.

In Vivo Antitumor Effect

MCF-7 cells (2×10^6) were dispersed in 100 μL PBS and were subcutaneously injected into the armpit of female BALB/c nude mice. After the tumor size reached approximately 100 mm^3, the mice were divided into four groups: PBS, LND, **FGLMC,** and **FGLMC** + Laser. The intravenous injection dose was 10 mg kg^{-1} each time for five times in total. The laser irradiation (660 nm, 100 mW cm^{-2} for 5 min) was given at 12 h post-injection. The tumor volume and body weights were measured every other day for 21 days after treatment (Tumor Volume = $W^2 \times L/2$, W = width, L =length). At the end of the efficacy study (day 21), all mice underwent surgery to remove tumors, and the main organs (heart, liver, lung, spleen, kidney) were collected for H&E staining. Mice survival was independently assessed for 30 days.

Statistical Analysis

The statistical analyses are shown as the mean ± standard deviation (SD) of the mean. Statistical significance (*$P < 0.05$, **$P < 0.01$, ***$P < 0.001$) was utilized by one-way analysis of variance (ANOVA).

Reference

1. Xu, G.; Zhang, H.; Wei, J.; Zhang, H.X.; Wu, X.; Li, Y.; Li, C.; Zhang, J.; Ye, J. Integrating the g-C_3N_4 nanosheet with B–H bonding decorated metal–organic framework for CO_2 activation and photoreduction. *ACS nano* **2018**, *12*, 5333–5340. https://doi.org/10.1021/acsnano.8b00110

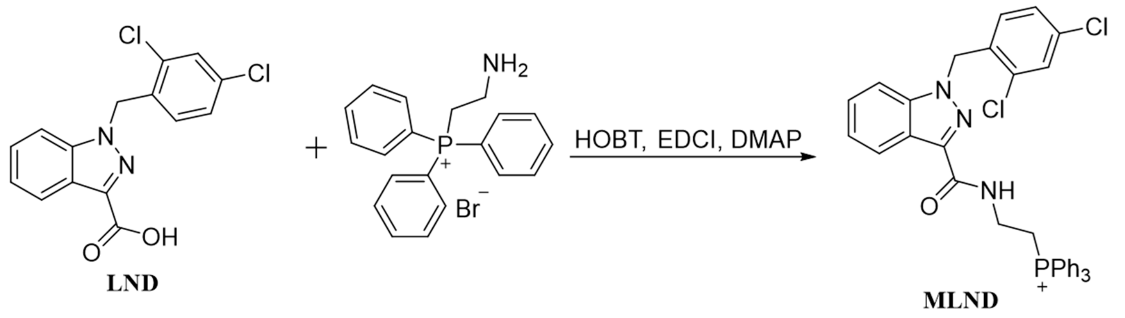

Scheme S1. Synthesis of MLND.

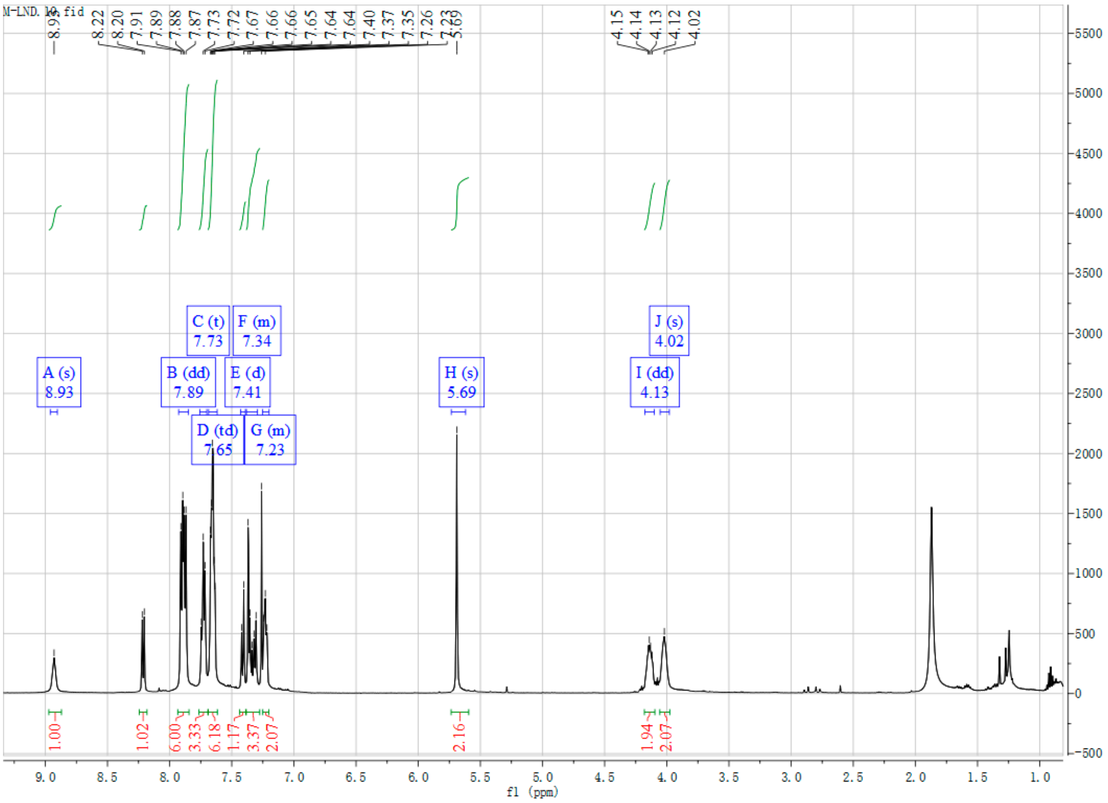

Figure S1. ^{1}H NMR of MLND.

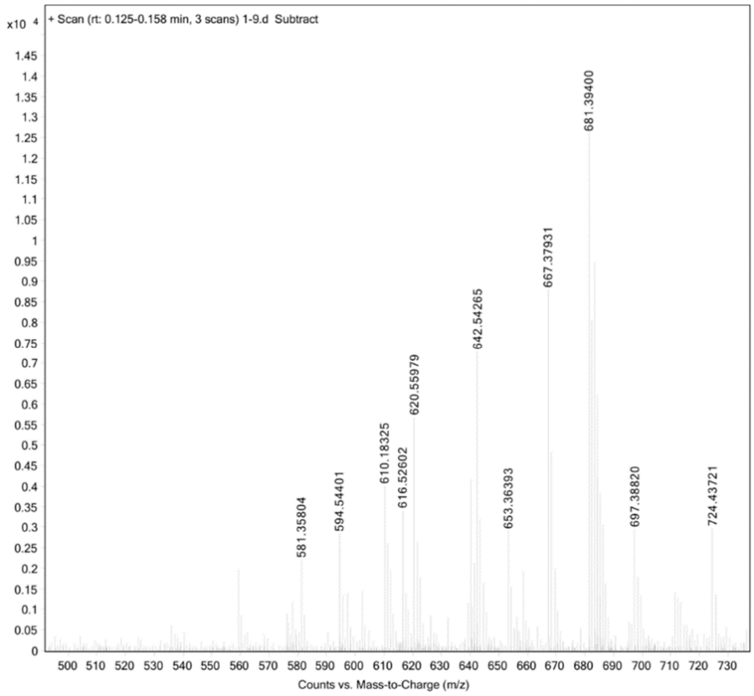

Figure S2. ESI-MS spectrum of MLND.

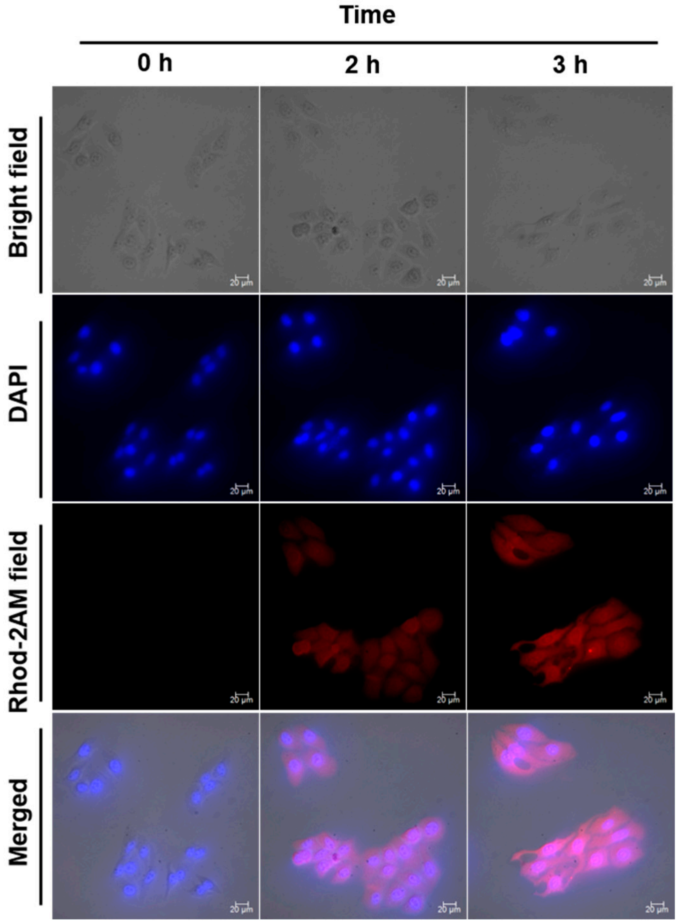

Figure S3. Calcium release of **FGLMC** with different incubation times (Scale bars represent 20 μm).

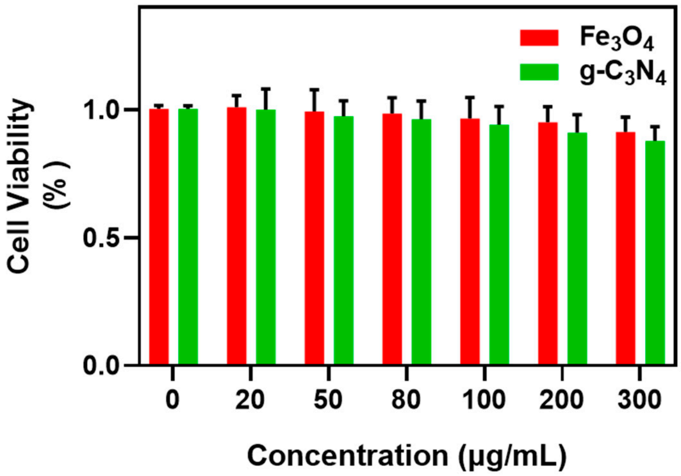

Figure S4. Cell viability of MCF-7 cells incubated with Fe_3O_4 and g-C_3N_4.

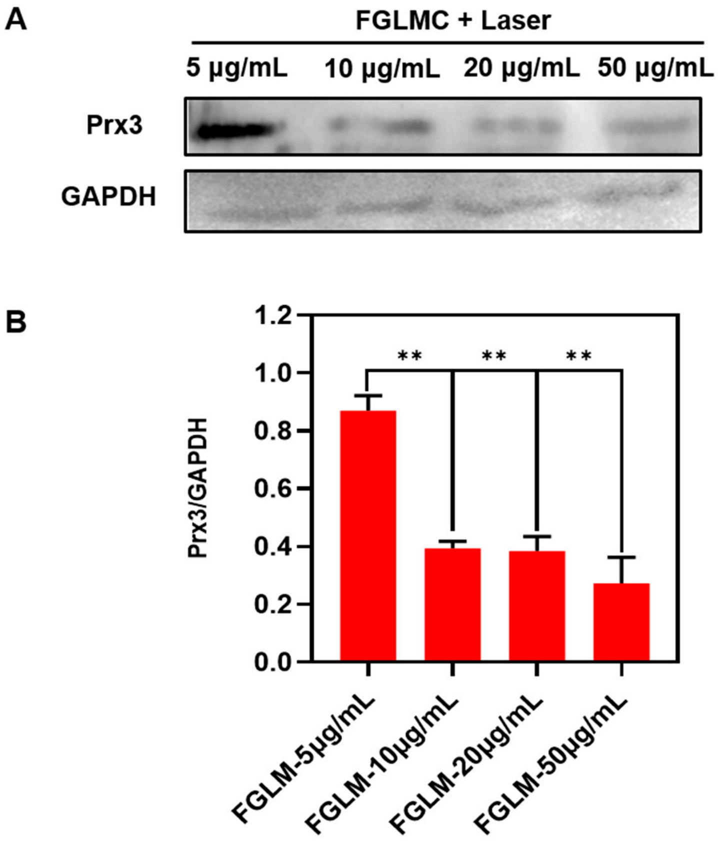

Figure S5. (A) Western blot analysis and (B) quantification for the expression of Prx3 on MCF-7 cells treated with different concentrations.

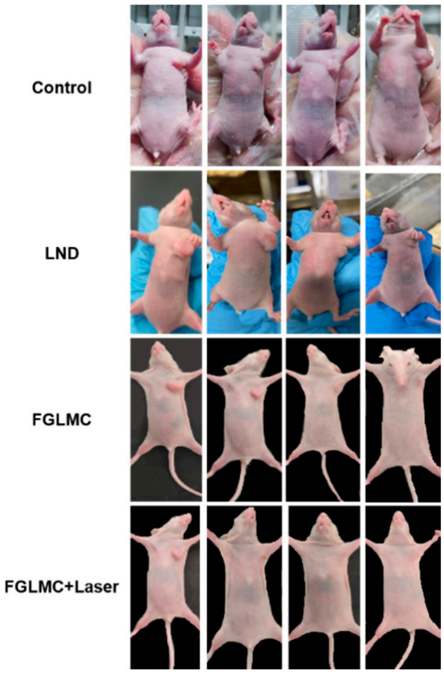

Figure S6. Digital photographs of MCF-7 tumor-bearing mice before and after different treatments.
